# Reduction of Oxidative Stress through Activating the Nrf2 mediated HO-1 Antioxidant Efficacy Signaling Pathway by MS15, an Antimicrobial Peptide from *Bacillus velezensis*

**DOI:** 10.3390/antiox9100934

**Published:** 2020-09-29

**Authors:** Md Maruf Khan, Young Kyun Kim, Tahmina Bilkis, Joo-Won Suh, Dae Young Lee, Jin Cheol Yoo

**Affiliations:** 1 Department of Pharmacy, College of Pharmacy, Chosun University, Gwangju 501-759, Korea; maruf.pharma@gmail.com (M.M.K.); arirangkyk1114@naver.com (Y.K.K.); 2 Department of Biomedical Sciences, Chosun University, Gwangju 501-759, Korea; bilkis.tahmina20@gmail.com; 3 Center for Nutraceutical and Pharmaceutical Materials, Myongji University, Myongji-ro 116, Cheoin-gu, Yongin 17058, Gyeonggi-Do, Korea; jwsuh@mju.ac.kr; 4 Department of Herbal Crop Research, National Institute of Horticultural and Herbal Science, RDA, Eumseong 27709, Korea; dylee0809@gmail.com

**Keywords:** peptide MS15, oxidative stress, ROS (reactive oxygen species), antioxidant, Nrf2/HO-1 (Nuclear factor erythroid 2-related factor-2/heme oxygenase-1)

## Abstract

The efficient culture and purification of antimicrobial peptides (AMPs), along with intense antioxidant activity, have drawn the interest to study antioxidant activity mechanism. We report the culture conditions optimization, efficient biosynthesis, and purification of an antioxidant peptide MS15 from *Bacillus velezensis* obtained from fermented food that would generate heme oxygenase-1 (HO-1) expression and lead to nuclear factor erythroid 2-related factor-2 (Nrf2) nuclear translocation. We explored the ability of kinetics and potency for the bacterial killing to work against various pathogenic bacteria. A bioassay showed the lysis zone of MS15 by tricine SDS-PAGE near at 6 kDa. MALDI-TOF/MS verified molecular weight, and the existence of a molecular mass of 6091 Da was reported by purity. The MIC of MS15 ranged from 2.5–160 μg/mL for many pathogenic bacteria, showing greater potency. In macrophage RAW 264.7 cells, MS15 was exposed to assess its inhibitory effect against the generation of reactive oxygen species (ROS) in oxidative stress. In the sample treated group, the translation, and transcriptional levels of CAT (catalase), GPx (glutathione peroxidase), and SOD (superoxide dismutase) were significantly greater. In short, MS15 has significant antioxidant properties, reducing ROS production in RAW 264.7 cells, and raising the translation and transcriptional rates of antioxidant enzymes with stimulating HO-1 induction facilitated by Nrf2.

## 1. Introduction

During aerobic metabolism, toxic ROS (reactive oxygen species) are produced, and final ROS production, including hydroxyl radicals (OH^•^), superoxide anions (O_2_^•−^), and hydrogen peroxide (H_2_O_2_), causes an array of chronic diseases, homeostasis imbalance, and oxidant stress [1]. ROS elevation was linked to the onset and progression of aging through oxidative damage and interaction with mitochondria [2]. Because of their reactivity, high ROS concentrations can induce oxidative stress by prooxidant balance and interrupting the antioxidant [3]. Rising research has shown that antioxidant peptide can enhance immune function and reduce oxidative stress [4]. Several studies are currently dedicated to discovering and using natural antioxidant peptide to remove unnecessary free radicals in individual health, therefore recognizing disease therapy and prevention [5]. The present research has proved that many diseases such as diabetes, Parkinson's disease, Alzheimer's disease, cardiovascular diseases, cataracts, arteriosclerosis, arthritis, and cancers, are strongly associated with cellular redox and free radicals imbalances [6]. The adoption of the natural antioxidant peptide is, therefore, the primary preference since natural peptide not only shows an essential function in the adjunctive treatment and prevention of diseases [7], it can also prevent unfavorable reactions to the individual physical condition.

Depending on their acting mechanism, antioxidant peptides have exhibited cellular protection counter to oxidative stress by the indirect or direct pathway [8]. Nuclear factor erythroid 2-related factor-2 (Nrf2) stimulated by a transcription factor initiated by leucine zipper redox-stress, allegedly is involved in cellular damage that mitigated producing oxidative stress [9]. The interaction between Keap1 (Kelch-like ECH-associated protein 1) and Nrf2 is disrupted during exposure to an antioxidant peptide that initiates Nrf2 and protein expression, thus enhances the mRNA of HO-1 (heme oxygenase-1), an antioxidant enzyme in cell lines [8]. The configuration alters of Keap1 to the nucleus over the interaction with various kinds of inducers releases Nrf2 translocate [10]. It combines with correlated antioxidant components in the cytoprotective gene promoter regions. Aerobic species have an efficient antioxidant ability to protect against oxidative stress, including vital enzymes such as catalase (CAT), glutathione peroxidase (GPx), and superoxide dismutase (SOD). Besides, detoxifying enzyme HO-1 (phase II) would induce through triggering Nrf2 [11].

Proteolytic microorganisms produce bioactive peptides derived from food sources that are commonly recognized as nutraceuticals for their functionality in disease prevention and in inhibiting the oxidative effect in food management. Species of *Bacillus* are mostly associated with enzymatic hydrolysis of amino acids and protein-producing peptides that appear to be beneficial to health [12]. Conventional fermented foods from *Bacillus* are regarded as a great source of protein and have been cultured in various countries [13]. The precise mechanism which causes antioxidant peptide activity was not fully known. However, numerous findings have demonstrated that those were lipid peroxidation inhibition, metal ion transition of chelators, and free radical scavengers [14]. Antioxidant properties of peptides are thought to be associated with their structural features, which include hydrophobicity, molecular weight, and amino acid sequences [15].

The current study aimed to isolate and purify a cytoprotective antioxidant peptide that acts against oxidative damage. To realize its activity counter to numerous pathophysiological disorders related to oxidative stress, we tried to verify by studying its antioxidant mechanism.

## 2. Materials and Methods

### 2.1. Reagents and Materials 

All chemicals employed for the investigational objective were of the highest analytical grade. Antioxidant reagents and lipopolysaccharide (LPS) were purchased from Sigma Aldrich (St. Louis, MO, USA). Bacterial strain CBSMS15 was obtained by the isolation process from Kimchi (traditional Korean food, local market, Gwangju, South Korea). DMEM (Dulbeco's modified eagle medium), Griess reagent (1% sulfanilamide in 5% phosphoric acid, 0.1% naphthylethylenediamine dihydrochloride), and DCF-DA (dichlorofluorescein diacetate) process have been used for experiments. RAW 264.7 macrophase cells were procured from ATCC (Rockville, MD, USA). Sephadex G-50 column and DEAE Sephadex A-50 column (Pharmacia, Uppsala, Sweden), and spin column (Thermo Scientific, Rockford, IL, USA) were used for purification. Tricine sodium dodecyl sulfate-polyacrylamide gel electrophoresis (tricine SDS-PAGE) and MADLI TOF/MS (matrix-assisted laser desorption/ionization time-of-flight mass spectrometer) (Ultraflex III, Burker, Germany) process were used to check the purification product confirmation. 

### 2.2. In Vitro Process of Isolation, Screening, and Culture Media Optimization 

The bacterial strain CBSMS15, which had a first nucleotide sequence similarity with *Bacillus* sp., was isolated from Kimchi. It could produce AMP (antimicrobial peptide), which was highly active against a wide range of pathogenic microorganisms. Kimchi is a distinctive typical fermented Korean food characterized by different microorganisms species such as *Lactobacillus* and *Bacillus subtilis*. We used cabbage kimchi for our study to isolate *Bacillus* strain. The isolation process was described briefly in our previous report [16], whereas 0.85% NaCl was mixed with 1 g of fermented kimchi sample and incubated overnight at 37 °C. Subsequently, the samples were serially diluted up to 10^−8^ in Mueller–Hinton (MH) broth. The proper CFU/mL (colony forming units) were assessed by streaking from those diluted aliquots. Hereafter, for the further stock, glycerol was used with appropriately diluted aliquots by 20% and 80%, respectively. The strain was discovered as *Bacillus* bacterial strain, based on morphologically characterized bacteria. It was analyzed following Bergey's manual of systematic bacteriology [17]. Moreover, the identification was established with the phylogenetic tree by 16S ribosomal RNA sequence analysis.

Antimicrobial activity was established by the method of disc diffusion. Briefly, paper disc filter (8 mm, Roshi Kaisha, Tokyo, Japan) soaked on the face of the petri dish with AMP (40 µL) was positioned, including the MH agar plate, overlay with *Mycobacterium* smegmatis [18]. Optimization of production culture media has enormous implications in the enhanced culture of AMP. Using de Man–Rogosa–Sharpe (MRS) media as control, culture medium optimization was brought out with different carbon, nitrogen, and metal ion sources [19]. In brief, numerous carbon sources (sucrose, maltose, sorbitol, mannitol, starch, fructose, glucose, lactose), nitrogen sources (beef extract, peptone, malt, oatmeal, yeast extract, tryptone, soybean meal), and metal ion sources (NaH_2_PO_4_, Na_2_HPO_4_, ZnSO_4_, MgSO_4_, KH_2_PO_4_, MgCl_2_, NaCl, FeSO_4_, CaCl_2_) were utilized in various amounts. Culture optimization was accomplished with diverse nitrogen sources (1%), and carbon sources (1%), with numerous metal ion sources (0.01%) till the last optimization. Finally, a metal ion, nitrogen, and carbon source were utilized to verify the final percentage of culture media by response surface methodology.

### 2.3. Optimizations of Culture Media Design by Response Surface Methodology and Statistical Analysis 

A response surface optimizer was applied to verify the optimum environments for soluble starch as carbon sources, yeast extract as nitrogen sources, and NaCl as metal ion sources compositions [19]. Design-Expert software (Stat-Ease Inc, Minneapolis, MN, USA) version 12.0 was used to verify the percentages of different sources by Box-Behnken method design. Each variable, denoted A, B, and C were assimilated. Every single factor can be evaluated, down level (−1), midpoint (0), and up level (+1), at three different levels, while the dependent variable was for bacteriocin activity (AU/mL) (Table 1). Totally 17 individual experimental runs were presented in a fractional factorial design. The program offered a prediction of bacteriocin activity (AU/mL). The probability levels above 95% (* *p* ˂ 0.05) discovered the effect of every single considerable variable on the activity of bacteriocin. 

### 2.4. Culture and Purification of Peptide MS15 

Seed culture produced on De Man, Rogosa, and Sharpe (MRS) media was continuously shaken at 160 rpm for 12 h at 37 °C. The purification process was staged for the culture of peptide MS15 for 32 h at 37 °C, and shaking speed was 170 rpm, including 1.5% soluble starch, 1% yeast extract, 0.01% NaCl obtained by response surface methodology. Then, cell-excluded supernatant was accumulated after centrifugation (10,000× *g*) (supra 22K, Hanil Sci. Ind., Incheon, Korea) for 30 min at 4 °C. Active precipitation fraction was recovered by mixing diammonium sulfate at the saturation of 20–80% using centrifugation at 10,000× *g* at 6 °C for 50 min. Aliquots dialyzed with 10 mM Tris-HCl (pH 7.2) buffer and subjected to ultrafiltration (Millipore co., Billerica, MA, USA) with 1 kDa and 10 kDa membrane. Following dialysis and filtration, the biologically active fraction was applied to a Sephadex G-50 (3 × 90 cm) column. The active fractions were combined and employed to ion-exchange separation using the DEAE Sephadex A-50 column (1.2 × 35 cm) and eluted, including the equal buffer. Further, active aliquots were utilized to a spin column named the Pierce spin desalting column and were investigated for purity check. 

### 2.5. Electrophoresis with Tricine SDS-PAGE and Bioassay (In Situ) Analysis 

The protein content was assessed using BSA (bovine serum albumin) as a reference protein by the Bradford method [20]. Tricine SDS-PAGE determined the molecular mass of the peptide [21]. The peptide was calculated with molar mass and purity employing Tricine SDS-PAGE. The bioassay was achieved alongside the designator organism (5.5 × 10^7^ CFU/mL) by covering the treated gel [22]. The gel was washed with 50 mM Tris-HCl buffer (pH 7.5) from tricine SDS–PAGE, consisted of 2.5% Triton X-100 for numerous times on 0.6% agar on Mueller–Hinton (DIFCO, San Diego, CA, USA) covering the reference organisms media and kept at 37 °C.

### 2.6. MALDI-TOF/MS for Molecular Weight Determination 

The MALDI-TOF method was utilized to check the molecular weight of peptide MS15 as well as the purity check of the final peptide sample [23]. To assess the process, we used the Ultraflex III (Burker, Germany). The evaluations were performed with a mass spectra range of *m*/*z* 500–40,000.

### 2.7. pH and Thermal Stability of MS15 

By investigating the temperature stability of MS15, aliquots were subjected to different treatments of temperature 0–100 °C and 121 °C for 60 and 15 min, respectively, before assessing the residual activity. The specimens were evaluated aligned with the *Escherichia coli* KCTC 1923 predictor microbial strain. Likewise, a large variety of buffers such as sodium phosphate-citric acid (pH 4.2–6.5), Tris-HCl (pH 7.2–9.4), and KCl-NaOH (pH 10.8–12.4) were tested for pH stability. 

### 2.8. Evaluation of Antimicrobial Susceptibility 

The agar dilution method utilized to test the minimum inhibiting concentration (MIC) of peptide MS15 was used to assess the antimicrobial inhibitory range [24]. Vancomycin and Bacitracin were carried as standard antibiotics comparing MS15 MIC value. Pathogenic microorganisms that contain specific concentrations of peptide MS15 and reference antibiotics were added to the MH-agar medium, and incubation was performed at 37 °C. MIC was distinguished according to the concentration of the dilution, which could diminish selected microorganism's visible growth (1.0 × 10^7^ CFU/mL). Gram-negative bacteria: *Salmonella typhimurium* KCTC 1925, *Pseudomonas aeruginosa* KCTC 1637, *Extended-spectrum beta-lactamase* W_1_, *Alcaligenes faecalis* ATCC 1004, ESBL 31, *Escherichia coli* KCTC 1923, ESBL V4 *(Escherichia coli)*; Gram-positive bacteria: VRE 4, VER 89, VRE98, *Staphylococcus aureus* KCTC 1928, *Mycobacterium smegmatis* ATCC 9341, *Bacillus subtilis* ATCC 6633, *Enterococcus faecalis* ATCC 29212, *Micrococcus luteus* ATCC 9341, MRSA B15, VRSA, MRSA 5-3, MRSA B15. The broth dilution method was employed to get the minimum bactericidal concentration (MBC) value [25]. MBC has been rated as the lowest concentration to inhibit bacterial growth fully (≥99.9%). 

### 2.9. Bacterial Killing Kinetics Assay of Peptide MS15 

Bacteriocin activity of MS15 was studied in the logarithmic growth phase (Log_10_), in time-kill kinetics findings analyzed with *Escherichia coli* cells (5.5 × 10^5^ CFU/mL), which was prepared in LB broth medium [26]. Various concentrations of MS15 (half of MIC to four times of MIC; 1 mL) was inserted into 10 mL LB medium and incubated at 37 °C with different time intervals of 0 to 36 h. The CFU (colony-forming unit) of *Escherichia coli* cells was established. The GC (growth control) study was presented without peptide for the species. The logarithm graph (CFU/mL) was designed alongside the time of growth.

### 2.10. DPPH (2,2-Diphenyl-1-Picrylhydrazyl) Radical Scavenging Capability Assay

The scavenging capacity of DPPH radical was evaluated, applying the procedure portrayed by Nanjo et al. with a minor modification [27]. A 100 µL various concentration (2.5–20 µM) of peptide MS15 sample solution (ethanol itself acted as a control) was vigorously mixed to DPPH (100 µL; 50 µM) in ethanol. Before the assay, the combination was left to keep in the dark place for 30 min at a normal temperature. Hereafter, the optical density of the final solution was assessed at 517 nm. Furthermore, ascorbic acid (2–16 µM) was evaluated as a reference antioxidant compound. The aptitude toward scavenging the DPPH radical was analyzed as:

$$\mathrm{Scavenging}\;\mathrm{activity}\;\mathrm{of}\;\mathrm{DPPH}\;\mathrm{radical}\;\left(\%\right)=\left[\frac{\left(A_{\mathrm{cont}}-A_{\mathrm{samp}}\right)}{A_{\mathrm{cont}}}\right]\times 100,$$

where A_samp_ is the optical density of the test sample and A_cont_ is the optical density of the blank (control). All analyses were studied in triplicate.

### 2.11. ABTS (2,2′-Azino-bis (3-Ethylbenzthiazoline-6-Sulfonic Acid)) Radical Scavenging Capability Assay

The ABTS^•+^ radical decoloration action method was applied to determine the ABTS^•+^ radical scavenging capability of peptide MS15 with a slight modification of the method portrayed by Carrasco-Castilla et al. [28]. The stock solution was formulated with ABTS (7 mM) and 2.45 mM potassium persulphate, which was made in water and left in the dark for 14 h to proceed with radical growth. Furthermore, the ABTS^•+^ solution was resuspended in ethanol and equilibrated to offer an absorbance of 0.705 ± 0.04, which was achieved at 734 nm. Variable concentrations (2.5–20 µM) of the peptide fraction (10 µL) were let to mix with 990 μL of the ABTS^•+^solution for 5 min and the optical density was noted at 734 nm. Ascorbic acid (2–16 µM) was analyzed as a reference standard antioxidant molecule. The % (percentage) inhibition of the ABTS^•+^ radical was evaluated as:

$$\mathrm{Scavenging}\;\mathrm{activity}\;\mathrm{of}\;{\mathrm{ABTS}}^{\;•+}\;\mathrm{radicle}\;\left(\%\right)=\left[\frac{\left(A_{\mathrm{cont}}-A_{\mathrm{samp}}\right)}{A_{\mathrm{cont}}}\right]\times 100,$$

where A_samp_ is the optical density of the sample and A_cont_ is the optical density of the control. All analyses were studied in triplicate.

### 2.12. Superoxide Radical Scavenging Capability Assay 

The capability assay of superoxide anion radicals was checked in the riboflavin/EDTA system by UV exposure [29]. The reaction combination consisting of DMPO (0.1 M), EDTA (5.0 mM), riboflavin (0.3 mM), and various concentration of MS15 dilutions were exposed under a UV lamp for 60 s at 365 nm. A mixture of the reaction was transported through a sealed capillary tube into the ESR spectrometer cavity, and the spin adduct was reported.

### 2.13. FRAP (Ferric Reducing Antioxidant Power) Activity Assay 

The FRAP assay was brought with a minor alteration for the measurement of reduction power, as previously described by Benzie et al. [30]. In brief, the FRAP reagent included, 10 mM 2,4,6-Tris(2-pyridyl)-s-triazine (TPTZ) solution in HCl (40 mM), ferric chloride solutions (FeCl_3_·6H_2_O; 20 mM), and acetate buffer (pH 3.5; 300 mM) with a ratio of 1:1:10. A 10 μL aqueous sample was mixed at different concentrations and 990 μL of FRAP mixture and incubated at 37 °C for half an hour. The optical density was assessed at 595 nm. Besides, ascorbic acid as a standard antioxidant compound was also studied.

### 2.14. CUPRAC (Cupric Reducing Antioxidant Capacity) Activity Assay 

CUPRAC assay of MS15 was established using the process explained by Apak et al., with minor modifications [31]. A solution of ammonium acetate buffer (1 M; pH 7.2), neocuproine (7.5 mM), and CuCl_2_ (10 mM), and the aliquots were combined to the ultimate solution. After one hour of incubation at 37 °C, the optical density was measured at 450 nm. Moreover, ascorbic acid was employed as a reference antioxidant compound.

### 2.15. ORAC (Oxygen Radicle Absorbance Capacity) Assay 

Following the previous article [32], the ORAC assay was executed. Trolox was employed as a positive control, which was an analog of vitamin E that was a water-soluble compound. After adding 20 mM AAPH with an emission wavelength of 520 nm and excitation wavelength of 480 nm, the analyzer was designed to take absorbance of the fluorescence of 200 nM fluorescein in a minute. The test was performed under pH 7.5 conditions at 37 °C with a blank sample in similar. The findings were determined in the areas under the fluorescence decay curves, applying experimental and blank sample variations stated as the Net AUC (area under the curve) values.

### 2.16. Cellular NO (Nitric Oxide) Measurement in RAW 264.7 Cells 

Griess reagent was utilized to calculate the concentration of nitric oxide (NO) in the medium as an indicator of NO output as our previous report [33]. At a count of 1.8 × 10^5^ cells per well, RAW 264.7 cells were inoculated into a 96-well microplate and incubated at 37 °C for 1 h. At different concentrations of MS15 (2.5–20 µM), the cells were then treated with 1 µg/mL LPS (lipopolysaccharide). NO concentration of the supernatants was assessed with the addition of Griess reagent after 24 h incubation. The absorbance of the mixtures was assessed at a wavelength of 520 nm, employing a fluorescence microplate scanner.

### 2.17. Intracellular ROS (Reactive Oxygen Species) Measurement in RAW 246.7 Cells 

The DCF-DA method was applied to determine the cellular oxidative pressure produced by LPS because of reactive oxygen species (ROS) [34]. The amount of ROS formed was associated with fluorescence intensity. RAW 264.7 cells were first inoculated with DMEM in 96-well plates (1.8 × 10^5^ cells/well) for 24 h. Various concentrations of MS15 (2.5–20 µM) subsequently pretreated in the cells after incubation and incubated for one hour. Moreover, cells were activated by LPS (1 µg/mL) and set for an extra 24 h. The cells were resolute two times with phosphate-buffered saline (PBS) buffer and treated with DCF-DA (25 μM) in a dark place at 37 °C for 30 min. Gallic acid (1.5–18 µM) was analyzed as a reference standard antioxidant compound. The fluorescence intensity was assessed employing a fluorescence microplate reader at an emission and excitation wavelength of 520 nm and 485 nm, correspondingly.

### 2.18. Cell Culture and Cell Cytotoxicity Assay 

Cells (RAW 264.7 cells and HeLa cells) were cultured in DMEM medium supplied with FBS (10%) and penicillin/streptomycin (100 µg/mL) at 37 °C under CO_2_ as our earlier report [35]. For cytotoxicity assay, cell seeding was performed into 96-well microplate at a density of 5.5 × 10^5^ cells/well (Nunc, Roskilde, Denmark). It was cultured and treated with various concentrations of MS15 according to the treatment plan. After incubation, 20 µL MTT (3-(4,5-dimethylthiazol-2-yl)-2,5-diphenyltetrazolium bromide) reagent (5 mg/mL) was put in to each well. After two hours of incubation, 100 µL of DMSO was dissolved and incubated for 12 h. Absorbance was noted using a microplate reader (KisanBio, Seoul, South Korea) at 570 nm.

### 2.19. Western Blot Analysis with Cell Lysates 

Cell lysates of RAW 264.7 cell were added with sample buffer (Tris-HCl (250 mM; pH 6.8), 2-mercaptoethanol (5%), glycerol (50%), bromophenol blue (0.5%), SDS (10%), DTT (0.5 M)), and boiled at 100 °C for 5 min to denature the mixture portrayed in the earlier study [36]. ReadyPrep^TM^ protein extraction kit (cytoplasmic/nuclear) from Bio-Rad (Bio-Rad Lab., Hercules, CA, USA) was used to extract nuclear protein. Protein aliquots (20 µg) were separated using SDS-PAGE (10%) and electrotransfer to polyvinylidene fluoride membranes (Millipore, Bedford, MA, USA). Hereafter, the membranes were incubated with primary antibodies in skim milk (5%). A series of primary antibodies named β-actin, anti-Nrf2, anti-HO-1, and lamin B were utilized. Horseradish peroxidase (HRP) (Santa Cruz Biotech. Inc., Santa Cruz, CA, USA) was used as secondary antibodies. The ECL (electrochemiluminescence) solution system (Perkin Elmer) was applied to discover the antibody–antigen reaction. 

### 2.20. RT-PCR (Reverse Transcription-Polymerase Chain Reaction) Assay 

TRIzol reagent (Invitrogen Co., Carlsbad, CA, USA) was used to extract total RNA from RAW 264.7 cells by following our prior study [33]. Total RNA (5 µg) was transcribed employing RT& GO Mastermix (MP Biomedicals, Seoul, Republic of Korea) into cDNA, and the product was applied to the PCR thermal cycler. Oligonucleotide primers (*SOD1*, *CAT*, *Nrf-2*, *GPx-1*, *GAPDH*, *HO-1*) were amplified with a denaturation step.

Sequences of the primer designed for RT-PCR were stated as *SOD1* (NM_011434.2): forward, 5′-CAG CAT GGG TTC CAC GTC CA-3′; reverse, 5′-CAC ATT GGC CAC ACC GTC CT-3′; *CAT* (NM_001752.4): forward, 5′-AG GCT CAG CTG ACA CAG TTC-3′; reverse, 5′-GC CAT TCA TGT GCC GAT GTC-3′; *Nrf-2* (NM_010902.4): forward, 5′-CTT TAG TCA GCG ACA GAA GGAC-3′; reverse, 5′-TCC AGA GAG CTA TTG AGG GACT-3′; *GPx-1* (NM_001329503.2): forward, 5′-TTC CCG TGC AAC CAG TTTG-3′; reverse, 5′-GGA CGT ACT TGA GGG AAT TCA GA-3′; *GAPDH* (NM_001357943.2): forward, 5′-GCG AGA TCC CGC TAA CAT CA-3′; reverse, 5′-AGT GAT GGC ATG GAC TGT GG-3′; *HO-1* (XM_002743721.5): forward, 5′-CCA GAA AGT GGG CAT CAG CT-3′; reverse, 5′-GTC ACA TTT ATG CTC GGC GG-3′. Ethidium bromide staining was used to visualize the PCR products after electrophoresis. 

### 2.21. Statistical Analysis 

The experimental data were presented with three replications per inspection, and every single point signifies the mean ± SD (standard deviation). Statistical analysis was completed employing one-way predictive analysis ANOVA (IBM SPSS statistics software; version 20.0). Difference was measured significant in statistics among the treatment represent as, * *p* < 0.05, ** *p* < 0.01, *** *p* < 0.001.

## 3. Results

### 3.1. Strain Isolation and Identification

**Figure 1:**
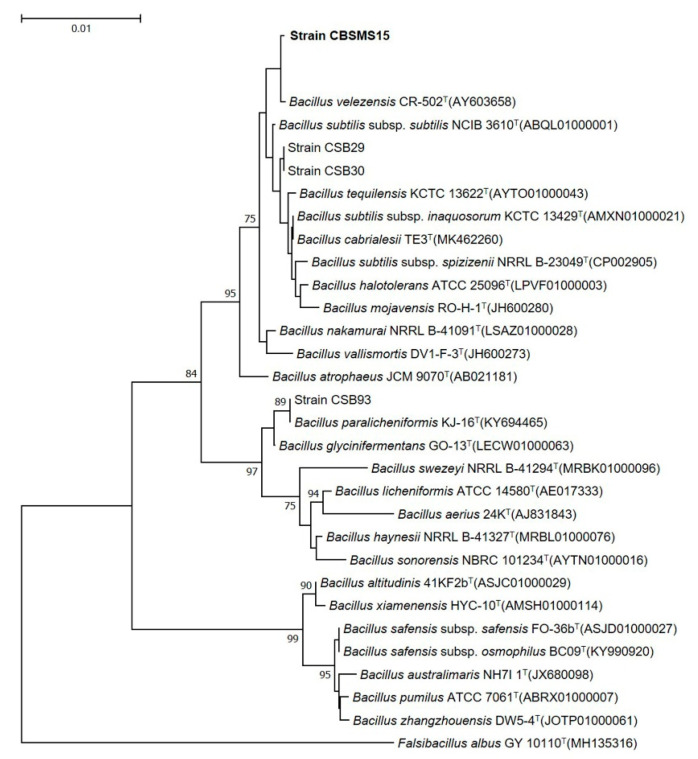
Phylogenetic tree, established on a full sequence of 16S rRNA genes, indicating the correlation among the CBSMS15 strain and closely linked *Bacillus*-type taxa. Node numbers are percentage bootstrap values based on 1000 replicates above values. The bar reflects 0.01 replacements by nucleotide type.

Microbial strains that were obtained and isolated from Kimchi (traditional Korean food) that have the capability of making AMP was preserved. Strain CBSMS15 was initially discovered based on its morphological and biochemical nature, and according to the results, the strain exhibited a close identity with some *Bacillus* strains with 16S rRNA gene sequences analysis. The 16S rRNA analysis showed the most intimate nature to *Bacillus velezensis* CR-502^T^ (Accession no. AY603658) with a homologous resemblance of 99.9%. A phylogenetic tree was shown in Figure 1, built from the 16S rRNA sequence evaluation. 

### 3.2. Experimental Design and Box-Behnken Analysis by Response Surface Methodology

Following the RSM theory, the higher value derived from the degree of the independent amount of choice, soluble starch (A), yeast extract (B), and NaCl (C) were additionally inspected at three distinctive levels. The bacteriocin production was substantially different in the medium from 7622 to 12367 AU/mL at varying levels of multiple elements (Table 2). 

**Figure 2:**
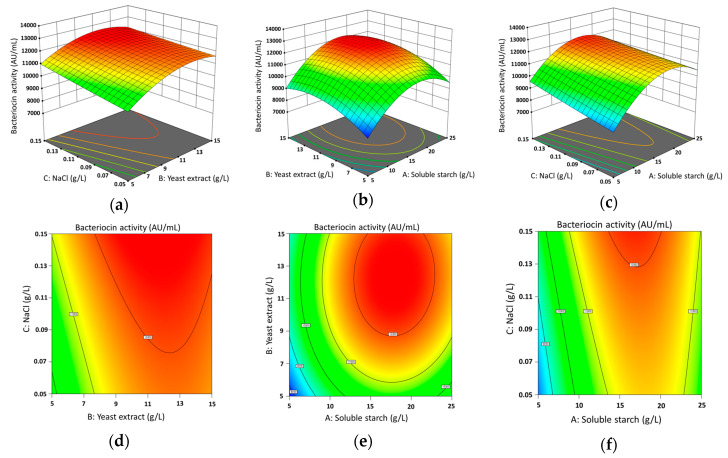
The plot of the system (3D) for response surface methodology showing the mutual and distinct effects of the variables on Bacteriocin activity (AU/mL). (**a**,**d**) optimized bacteriocin activity with the interaction between of yeast extract (g/L) and NaCl (g/L), (**b**,**e**) optimized bacteriocin activity with the interaction between of soluble starch (g/L) and yeast extract (g/L), (**c**,**f**) optimized bacteriocin activity with the interaction between of soluble starch (g/L) and NaCl (g/L).

The predicted median was noted as 11359.9 AU/mL ANOVA study had measured the fitness of bacteriocin activity. Analyzing data with ANOVA, the linear research of independent variables A, B, C, and cross term BC (Figure 2a), AB (Figure 2b), AC (Figure 2c) in bacteriocin activity was found to be statistically significant (*** *p* < 0.001; F-value 31.18). 2D dimensions were produced with cross term BC (Figure 2d), AB (Figure 2e), and AC (Figure 2f). After data assessment, we initiate that the interface of AB, BC, and AC interactions corresponding to the highest bacteriocin activity peak at 13.424 g/L soluble starch, 7.636 g/L yeast extract, and 0.089 g/L NaCl (Figure 2). The final equation in periods of essential components for bacteriocin activity was established. The comparison in terms of real considerations can be employed to create estimates regarding the reply for provided levels of every element. For each factor, the levels should be defined at this moment in the initial components.

Bacteriocin activity = −151.987 + 681.670 A + 879.920 B + 19,782.000 C + 1.930 AB − 492.000 AC − 680.000 BC − 18.466 A^2^ − 34.586 B^2^ + 9740.000 C^2^.

Therefore, focus on the model maximizing the adjusted coefficient determination, R^2^ showed 0.9857, and the predicted R^2^ showed 0.9444, which is near to 1.00, which improved the model.

### 3.3. Culture Media, Purification, and Molecular Weight Resolve of MS15

**Figure 3:**
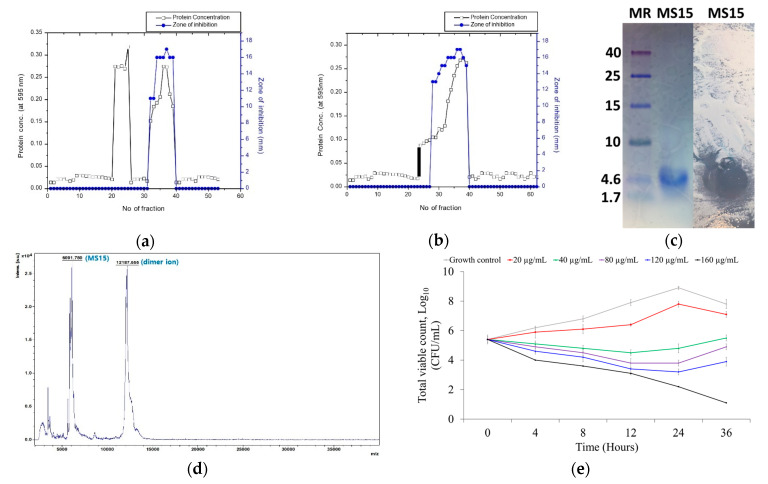
The elution profile of peptide MS15 from (**a**) the Sephadex G-50 column (3 × 90 cm) and (**b**) the DEAE Sephadex A-50 column (1.2 × 35 cm). (**c**) Tricine SDS-PAGE gel electrophoresis and bioassay (in situ); Lane 1: protein marker (low range protein ladder), Lane 2: purified peptide MS15, Lane 3: in situ analysis showed clear inhibition zone in the same area in the right side picture. (**d**) MALDI-TOF/MS method is used to obtain the molecular weight of purified MS15. (**e**) Bacterial kill kinetics with various concentrations of MS15 against *E. coli* for 36 h.

The maximum bacteriocin activity was achieved by culture media containing 1.342% soluble starch, 0.763% yeast extract, and 0.0089% NaCl and cultured for 32 h at 37 °C with 150 rpm. Peptide MS15 was purified from the culture supernatant and fractioned with the salting-out process. Two-step chromatography with size exclusion chromatography (Figure 3a) and anion exchange chromatography (Figure 3b) leads to a recovery of 5.92% activity with 24.49-fold and 16,000 AU total activity (Table 3). Clear, single, and purified peptide band observed by Tricine SDS-PAGE to a molecular weight of 6091 Da and lysis zone (bioassay) was showed within the same area where it showed the single pure peptide band against a reference bacteria *Mycobacterium smegmatis* (Figure 3c). 

### 3.4. Mass Spectroscopy Analysis by MALDI-TOF-MS

Purified MS15 were investigated primarily on a MALDI-TOF-MS that linear/reflector mode was applied. The analysis was done with linear mode (2–200 kDa) and reflector mode (500–6000 Da) to detect the purity and molecular weight of MS15. The molecular weight was at the peak point of 6.091 kDa for peptide MS15 (Figure 3d) that demonstrated a major peak of the peptide (n + 1).

### 3.5. Stability Analysis of MS15

Residual activity was examined corresponding to the preserved bacteriocin activity to verify the temperature stability of MS15, and it persisted in being stable up to 60 °C. However, its activity was declining slowly and retained about 84% of the bacteriocin activity after 30 min of incubation at 70 °C. Total loss of activity appeared for 15 min at 121 °C when it got exposure to autoclave conditions. The capability to maintain the MS15 activity in a wide selection of pH values can result in the denaturation of the variable protein. The peptide MS15 was incubated at various pH buffers to evaluate the stability of the pH. MS15 has been found to tolerate an extensive range of pH values (4.2–12.4). MS15 exhibited a wide pH and temperature array. 

### 3.6. Assessment of Antimicrobial Susceptibility and Time-Kill Kinetics Analyses

The MICs of peptide MS15 were assessed against different pathogenic bacteria, such as MIC of Gram-positive pathogens (2.5–160 µg/mL) and MIC of Gram-negative pathogens (MIC of 40–160 µg/mL). The concentration of MS15 concerning MIC was contrasted to the value of MIC, which represents the standard antibiotic of bacitracin and vancomycin (Table 4). Antimicrobial activity of peptide MS15 was more potent rather than the standard antibiotic counter to a broad array of pathogenic bacteria, which is different from Gram-positive pathogen, for instance, Vancomycin-resistant *Enterococci* 98, Vancomycin-resistant *Staphylococcus aureus*, Methicillin-resistant *Staphylococcus aureus* 5-3, *Staphylococcus aureus*, Vancomycin-resistant *Enterococci* 89, Methicillin-resistant *Staphylococcus aureus* B15, and Gram-negative pathogens, for instance, *Escherichia coli*, *Pseudomonas aeruginosa*, *Salmonella typhimurium*, *Extended-spectrum beta-lactamase* (W1, 31). The bactericidal activity was assessed by using the broth dilution method, and it kills bacteria in a range concentration of 5–320 µg/mL. 

The time-killing kinetics of peptide MS15 at different MIC concentrations indicated a decline in the total of viable cells around different time intervals, subsequently up to the 36 h against the reported organism *Escherichia coli*. A logarithm of viable colony count was analyzed with time intervals on multiple concentrations. Peptide MS15 decreased *Escherichia coli* count at 40 µg/mL (concentration of MIC) up to 12 h. An initial decrease in the amount of surviving *Escherichia coli,* at 40 µg/mL peptide concentrations was detected. *E. coli* cells recovered from the peptide activity after 12 h remaining and began to develop again. Mostly regrowth was noticed up to 120 µg/mL, although 160 µg/mL demonstrated a total decline of the *E. coli* count (Figure 3e).

### 3.7. Radical Scavenging Activity Assay

**Figure 4:**
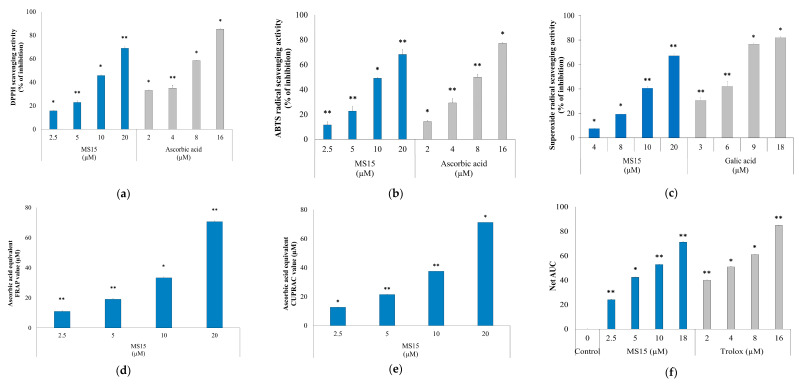
(**a**) The DPPH, (**b**) ABTS^•^^+^, and (**c**) SOD radical scavenging assay were performed with several concentrations. (**d**) FRAP assay and (**e**) CUPRAC assay were conducted with various concentrations of MS15, where ascorbic acid and gallic acid were assessed as a standard antioxidant compound. (**f**) The net area under the curve (Net AUC) was obtained by subtracting the area under the blank curve from the area under the sample curve, which showed the ORAC properties of the samples. Applying the student’s *t*-test, significantly different from the control denoted as, ** *p* ˂ 0.01, * *p* ˂ 0.05.

ABTS^•^^+^ and DPPH radical-scavenging activity was significantly influenced (* *p* ˂ 0.05) by the concentration variation of peptide MS15 and reference standard ascorbic acid. Peptide MS15 exhibited concentration-dependent antioxidant activity in diverse in vitro antioxidant assay, containing scavenging of ABTS^•^^+^ and DPPH radicles. The results of the scavenging of DPPH and ABTS^•^^+^ were compared with reference standard ascorbic acid. In contrast, the constant free DPPH radical receives hydrogen radical or an electron from antioxidant and diminishes to develop a steady non-radical form DPPH-H, converted to diphenyl-picrylhydrazine molecule through resulting yellow in color and decline in absorbance. As displayed in Figure 4a, peptide MS15 exhibited excellent DPPH radical scavenging activity towards higher concentration, which was near to that of ascorbic acid. Our peptide showed a DPPH radical scavenging range of 17–69% in a dosages range of 2.5–20 µM. In contrast, ascorbic acid showed a DPPH radical scavenging range of 32–85% in a dosages range of 2–16 µM. 

The ABTS^•^^+^ discoloration assay directly associates, including the significance of the scavenging capability of an antioxidant compound involving its hydrogen donating capacity, which was profoundly colored and was concluded by evaluating absorbance at 734 nm. Furthermore, in ABTS^•^^+^ scavenging assay, the ABTS^•^^+^ radicle was pre-formed by the oxidation process along with potassium persulfate, which could be decreased in the existence of hydrogen-providing antioxidants. Peptide MS15 showed ABTS^•^^+^ radical scavenging up to 68% in a concentration of 20 µM (Figure 4b). In contrast, ascorbic acid showed ABTS^•^^+^ radical scavenging up to 77% in a concentration of 16 µM. On the other hand, our peptide MS15 showed a superoxide radical scavenging range of 6–67% in a dosages range of 3–18 µM (Figure 4c). In contrast, ascorbic acid showed a superoxide radical scavenging range of 29–82% in a dosages range of 4–20 µM. Remarkably, peptide MS15 considerably scavenged both the ABTS^•^^+^ and DPPH superoxide radical in a concentration reliant approach.

### 3.8. Reducing Power Measurement Assay

We checked the electron-donating capacity by utilizing the method of FRAP and CUPRAC. The mainly FRAP method is a complete electron transfer process where antioxidants diminish ferric ions. We found great activity through the FRAP process, whereas the FRAP value is equivalent to ascorbic acid found up to 72.73% (*p* < 0.001) in a concentration of 20 µM (Figure 4d). Otherwise, the CUPRAC assay is used to check the transfer of n-electron reductant antioxidants. The CUPRAC value equivalent to ascorbic acid found up to 71.34% (*p* < 0.001) in a concentration of 20 µM (Figure 4e). Trolox value (net AUC) and dose-dependent MS15 have increased the findings suggested that MS15 (18 µM) showed similar antioxidant activity to 16 µM Trolox (Figure 4f).

### 3.9. Cell Viability Assay

**Figure 5:**
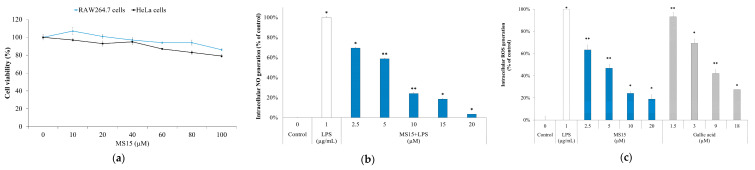
(**a**) Cytotoxicity assay, cell seeding (RAW 264.7 cells, and HeLa cells) was performed into 96-well microplate at a density of 5.5 × 10^5^ cells/well. (**b**) Generation of NO and (**c**) generation of ROS were evaluated in RAW 264.7 cells. Applying the student's t-test, a significant difference from the control denoted as, ** *p* ˂ 0.01, * *p* ˂ 0.05.

The MTT assay with various concentrations (10–100 µM) of MS15 was measured in RAW 246.7 cells, and human epithelial HeLa cells were thoroughly examined. The outcome demonstrated no cytotoxic effects at a concentration up to 40 µM, whereas the significantly less cytotoxic impact of up to 100 µM (Figure 5a).

### 3.10. ROS Measurement and Nitric Oxide Inhibitory Assay

ROS measurement and NO inhibitory assay were investigated in macrophage cell lines RAW 264.7, and results were compared with a reference standard antioxidant Gallic acid. The intracellular ROS level in macrophages proliferated in cells inspired through LPS or other inducements quickly increased, initiating oxidative stress. Hereafter, we attempted to assess MS15 antioxidant activity by analyzing the generation of NO and ROS. Stimulation of the LPS potentially enhanced the growth of the medium associated with control cells, which was untreated. In contrast, pretreatment with MS15 remarkably increased NO scavenging activity by 13.6% (*** *p* < 0.001), 33.2% (*** *p* < 0.001), 45.1% (*** *p* < 0.001), and 65.4% (*** *p* < 0.001) at dosages of 2.5, 5, 10, and 20 µM, correspondingly (Figure 5b). RAW 246.7 cells were evaluated by observing cell morphology to investigate other effects of MS15 on the ROS generation. The findings displayed that pretreatment with MS15 significantly reduced ROS generation by increasing concentration (Figure 5c). Gallic acid was presented as a positive control that demonstrated being active in reducing cellular ROS generation. These findings imply that both MS15 reduced the generation of NO and the generation of intracellular ROS deprived of any cellular toxicity.

### 3.11. Effects of MS15 on Antioxidant Enzyme in RAW 264.7 Cells

**Figure 6:**
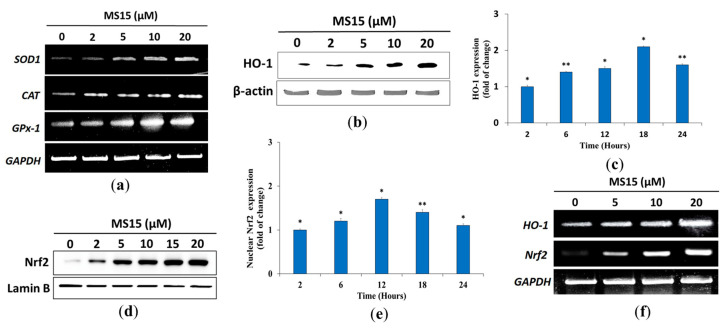
Effects of MS15 on the expression mRNA levels were checked on the detoxifying enzyme, primary and Phase II antioxidant in RAW 264.7 cells. RAW 264.7 cells with the required concentration of MS15 were pretreated for 24 h. (**a**) RT-PCR was used to test the mRNA expression of Phase II antioxidants, and primary antioxidant enzymes (GPx-1, CAT, SOD1) were assessed in a concentration reliant way. The expression of HO-1 was evaluated by Western blot analysis in (**b**) a concentration reliant and (**c**) time reliant (the fold of change) way. Impact of MS15 on Nrf2 activity, RAW264.7 cells with the required concentration of MS15 were pretreated for 24 h. Western blot analysis was used to calculate the nuclear translocation of Nrf2 in (**d**) a concentration reliant and (**e**) a time reliant (fold of change) way. (**f**) The mRNA of the detoxifying enzyme (HO-1) and Nrf2 were assessed by RT-PCR in a concentration reliant way.

In order to explore the impact of MS15 on antioxidant enzymes (GPx-1, CAT, SOD1) and detoxifying enzymes of phase II (HO-1), RT-PCR analysis was used to assess a concentration-dependent rise in the mRNA expression of GPx-1, CAT, and SOD1 (Figure 6a). RAW 264.7 cells were used to treat with MS15 at 2, 5, 10, and 20 µM for 24 h. Western blot assessments were used to confirm the protein levels of HO-1 (Figure 6b). Furthermore, the Western blot assessment (time reliant) showed substantially increased protein expression of HO-1 after the treatment of MS15 (Figure 6c). Nrf2 is typically attached to the Keap1 protein, and the stimulation of antioxidant enzymes (phase II) can be accomplished while Nrf2 was left off, and the nucleus was translocated. It was noted that treatment with MS15 increased the amount of Nrf2 mRNA in a dose reliant way (Figure 6d). The mechanism of activation of Nrf2 by MS15, along with the protein levels of Nrf2 translocation in the cytosol, was evaluated in a time-dependent enhancement process to explain after 24 h treatment (Figure 6e). MS15 therapy has been able to increase HO-1 transcriptional and translation rates. The transcription level of the Nrf2 was evaluated to verify that MS15 stimulates phase II enzymes via Nrf2. It was discovered that MS15 considerably enhanced Nrf2 mRNA expression (Figure 6f).

## 4. Discussion

The principal goal of the study was to purify a potential antioxidant peptide that has potent antimicrobial activity and evaluate its antioxidant activity pathway. In the current study, we described the impact of the different components of carbon, nitrogen, and metal-ion sources of several culture conditions and growth media to get optimum bacteriocin activity. Growth media composition, temperature, and pH of dissolved peptide played a crucial part in the production and stability of bacteriocin activity. Peptide MS15 imposed cyclic structure so that the amino acid sequence could not be detected by using the Edman degradation method, although purification was detected with MALDI TOF/MS. The peptide was purified by two-step column chromatography with the 24.49-fold and recovered activity of 5.92%, that of total bacteriocin activity was found at 16000 AU, which was consistent with our previous report [18,22]. Primarily, we investigated the antimicrobial spectrum of peptide MS15 against different pathogens that were significantly higher than our previous report [37]. In contrast, it showed better MBC value, produced high bacteriocidal activity, which can be compared with our previous article [22]. Time-kill kinetics findings showed a rapid decline and regrowth of *Escherichia coli* cells after 12 h, which is consistent with the kinetics of other antimicrobial peptide reports [38,39].

Oxidative stress is characterized as a discrepancy among free radical development and its removal by mechanisms of cellular defense. ROS consists of hydroxyl radical (OH^•^), superoxide anion (O_2_^•^), and hydrogen peroxide (H_2_O_2_) and are by-products of cellular metabolic effects, and pathways are intracellular, toxic organisms, partially formed by oxygen reduction (O_2_) [40]. ROS is well known to be chemically further reactive than O_2_^•^ and thus, ROS was assumed to perform primarily as cellular damaging agents, responding extensively to lipids and proteins [41]. From the study, antioxidant activity was assessed using different methods and the fundamental mechanism by which MS15 mitigated oxidative stress throughout the translation and transcriptional in RAW 264.7 cells via the Nrf2-Keap pathway. A compound is an antioxidant when it can stabilize, has an electron or hydrogen donating capability, and transfer the unpaired electron, and has the power to chelate metals [42]. The most common spectrophotometric are DPPH^•^ and ABTS^•^^+^ methods for evaluating the antioxidant potential of any molecule under study. Peptide MS15 exhibited a high ABTS^•^^+^ and DPPH^•^ scavenging activity. The finding revealed that scavenging activity was performed by transferring hydrogen reaction (DPPH assay) or electron transfer reaction (ABTS assay). As presented in Figure 4a,b, peptide MS15 demonstrated more active radical scavenging activity than its precursor, in contrast with earlier reports [43]. MS15 displayed a considerably superior quench of SOD radical as a dose reliant rise in inhibition percentage. 

The variation of color evaluated antioxidant power in the FRAP from the reduction of the yellow ferric complex to the blue ferrous complex. Otherwise, due to the smaller redox potential of CUPRAC reactive, citric acid was not oxidized, and sugar decline with the CUPRAC reactive. CUPRAC and FRAP are significant measures of reducing the ability of an antioxidant that is correlated with the earlier report [44] with the involvement of samples liable for separating the free radical chain by hydrogen atom donation. The findings showed the visible antioxidant ability of MS15, which was rising slowly with improving sample strength compared to ascorbic acid equivalent. Besides, the ORAC assay uses a peroxyl radical derived from LPS, which mimics the peroxyl radical of lipid that participating in the in-vivo chain reaction of lipid peroxidation. An antioxidant has a protecting impact which is evaluated by an assessment of the region covered by the curve of fluorescence decay (AUC) that had higher reduction capability [32]. 

The power of cellular antioxidants derives from their immediate power, described as the ability to quench free radicals, with either electrons or hydrogen contributing to ROS. Simultaneously, indirect capability includes defending alongside oxidant stress by stimulating detoxify the expression and antioxidant enzymes in Phase II [45]. During cell proliferation and maintenance, the scavenging activity of ROS performs a crucial part in cell homeostasis. A variety of SOD, GPx, and CAT enzymes are related to eliminating these free radical species in cells. If multiple occurrences of oxidative stress damage these enzymes, then progressive diseases may result. An electron O_2_ reduction forms the superoxide of cytosol (O_2_^−^) through the slipping of electrons from the electron transport chain of the mitochondrial electron carriers. It was entirely proven that SOD promptly transforms O_2_^−^ into H_2_O_2_. Besides, the antioxidant enzymes, GPx, and CAT, will detoxify H_2_O_2_. Those enzymes function collectively as free radicals in the metabolic pathway [46,47]. In this analysis, together with mRNA and protein levels of antioxidant enzymes, for instance, GPx-1, CAT, and SOD1 in RAW 264.7 cells, MS15 treatment enhanced radically, demonstrating the ability of MS15 to preserve the state of cell homeostasis and defend the cell against oxidative stress.

**Figure 7:**
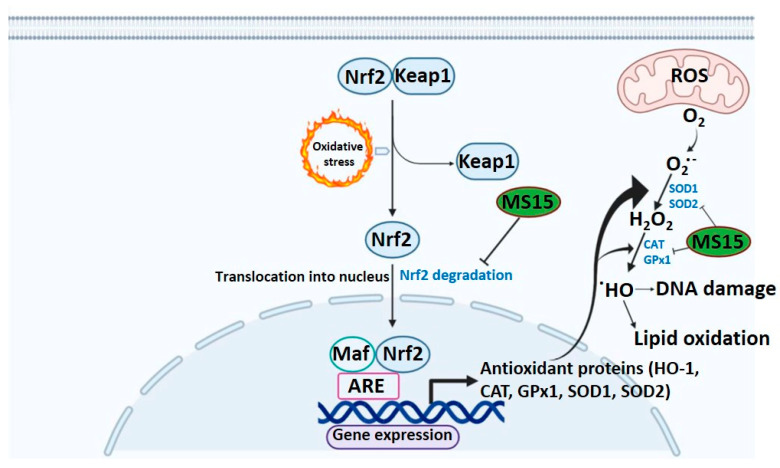
Illustration of hypothetical antioxidant mechanism for peptide MS15 against oxidative stress.

Under normal conditions, the Keap1 protein binds Nrf-2 in the cytoplasm and performs a crucial part in Phase II enzyme stimulation when released and translocated to the nucleus [48]. MS15 treatment has been competent to enhance the translational and transcriptional levels of HO-1 and, in line with previous therapies, facilitates the Nrf-2 translocation in a concentration-reliant way into the nucleus. The process suggests that the antioxidant properties of MS15 are partly due to the concentration-dependent initiation of HO-1 in RAW 264.7 cells, controlled by dose-reliant activation of Nrf-2 [49]. However, the inhibition of the HO-1 role by HO-1 inhibitor considerably hindered those protective impacts of MS15 against H_2_O_2_. Such findings are in the substantial arrangement among other latest results indicating that HO-1 reestablished cell persistence by preventing apoptosis induced by oxidative damage. The antioxidant role of HO-1 performs by turning heme into the strong biliverdin prooxidant and, ultimately, bilirubin, which is a powerful antioxidant [36]. The interface among MS15 and Nrf-2 that imitates the activity of other Nrf-2 inducers, such as caffeoylquinic acid and 5-O-sulforaphane, that antioxidant response element (ARE)-dependent gene expression in HT29 cells and modulates nuclear translocation on objectives, for instance, NQO-1, Nrf-2, and HO-1 [50]. The antioxidant mode of action of MS15 might be powered by ARE-dependent gene expression and nuclear translocation, given numerous suggested molecular procedures controlled by antioxidant peptides (Figure 7). 

Different synthesized antioxidant peptide analogs will investigate the antioxidant mode of action after elucidating the completed structural details of MS15 and assessing the subsequent findings, for instance, the phosphorylation of PI3K/Akt and MAPKs, and the luciferase assays. Thus, while further studies should be undertaken on the significance of several GSH-dependent enzymes, the present findings imply that MS15’s cellular protecting capacity against oxidative stress depends on, however, on the signaling pathway of Nrf2/HO-1 in RAW 264.7 cells.

## 5. Conclusions

The identification of novel antimicrobial and antioxidant peptide MS15 against oxidant damage, throughout its potential effects, might assist well in understanding its action against the various pathophysiological situations related to oxidant stress. This analysis clearly shows that MS15 has a presence of antioxidant operation, the capability to move hydrogen atoms, and the capacity to avoid oxidative stress caused by H_2_O_2_ in RAW 264.7 cells. It was done through increasing the amount of cellular thiol, GST activity, and antioxidant enzyme expression and redox-sensitive transcription factor Nrf-2 expression. In conclusion, the present study showed that peptide MS15, which was purified from *Bacillus velezensis*, had significant and dose-dependent antioxidant capacity. The proposed underlying mechanism through which MS15 mitigates oxidative stress via translational and transcriptional control of oxidoreductase (phase I) and detoxifying enzyme (phase II).

## Figures and Tables

**Table 1:** Experimental design and independent variables of choice for optimizing culture media.

Code	Variables of Choice (Independent)	Up Level	Midpoint	Down Level	Generated Optimum Factor
		(+1)	(0)	(−1)	
A	Soluble starch (g/L)	25	15	5	13.42
B	Yeast extract (g/L)	15	10	5	7.64
C	NaCl (g/L)	0.15	0.1	0.05	0.089

**Table 2:** Assessment of the generated data by the Box-Behnken experiment.

Run	Soluble Starch (g/L)	Yeast Extract (g/L)	NaCl (g/L)	Bacteriocin Activity of MS15 (AU/mL)	
	A	B	C	Actual Value ^x^	Predicted Value
1	15	10	0.1	12,138.00	12,205.80
2	5	15	0.1	9460.00	9166.00
3	15	10	0.1	11,940.00	12,205.80
4	5	10	0.05	8650.00	8782.25
5	15	5	0.05	10,450.00	10,072.25
6	5	10	0.15	10,113.00	10,029.25
7	25	10	0.05	11,146.00	11,229.75
8	15	10	0.1	12,268.00	12,205.80
9	15	5	0.15	11,329.00	11,167.25
10	25	5	0.1	9336.00	9630.00
11	5	5	0.1	7622.00	7867.50
12	15	10	0.1	12,316.00	12,205.80
13	25	15	0.1	11,560.00	11,314.50
14	15	15	0.05	11,742.00	11,903.75
15	15	15	0.15	11,941.00	12,318.75
16	25	10	0.15	11,625.00	11,492.75
17	15	10	0.1	12,367.00	12,205.80

^x^ The experimental data were presented by three replications per inspection. Different levels of three variables were used to get optimum culture conditions, whereas *** *p* < 0.001; F-value 31.18.

**Table 3:** Purification steps of peptide MS15.

Purification Steps	Vol (mL)	Total Protein (mg)	Total Activity (AU)	Specific Activity (AU/mg)	Purification Fold	Recovery (%)
Cell free crude sample	935	388.5	270,000	694.98	1	100
Ammonium sulphate pallet aliquots	52	98.45	156,000	1584.56	2.28	57.78
Sephadex G-50 gel	8	7.23	38,000	5255.88	7.56	14.07
DEAE Sephadex A-50 gel	2	0.94	16,000	17,021.27	24.49	5.92

All data represent the status of the sample after the designated procedure has been carried out. The purification fold was estimated based on starting crude sample activity.

**Table 4:** Antimicrobial field of activity for peptide MS15 against various pathogens.

Microorganisms	MIC of MS15 (µg/mL)			MBC of MS15 (µg/mL)
	MS15	Bacitracin	Vancomycin	
**Gram-negative bacteria**				
*Escherichia coli* KCTC 1923	40	160	80	80
*Pseudomonas aeruginosa* KCTC 1637	160	>160	>160	320
*Salmonella typhimurium* KCTC 1925	40	80	40	80
*Alcaligenes faecalis* ATCC 1004	160	160	80	320
*Extended-spectrum beta-lactamase* V4 *(Escherichia coli)*	80	160	80	80
*Extended-spectrum beta-lactamase* W1	40	80	40	80
*Extended-spectrum beta-lactamase* 31	40	80	40	80
**Gram-positive bacteria**				
*Vancomycin-resistant Enterococci* 4	160	80	160	320
*Vancomycin-resistant Enterococci* 89	80	>160	>160	160
*Vancomycin-resistant Enterococci* 98	80	>160	>160	160
*Staphylococcus aureus* KCTC 1928	160	>160	>160	160
Methicillin-resistant *Staphylococcus aureus* 5-3	2.5	5	2.5	5
Methicillin-resistant *Staphylococcus aureus* B15	40	160	80	120
*Mycobacterium smegmatis* ATCC 9341	20	40	2.5	40
*Micrococcus luteus* ATCC 9341	40	40	2.5	40
*Enterococcus faecalis* ATCC 29212	20	5	2.5	20
*Bacillus subtilis* ATCC 6633	10	20	0.8	20
*Vancomycin-resistant Staphylococcus aureus*	80	160	>160	160

MIC: minimum inhibitory concentration; MBC: minimum bactericidal concentration.
